# Sarcopenia in Kidney Transplantation: Bridging Pathophysiology to Patient-Centered Care

**DOI:** 10.3390/nu18091352

**Published:** 2026-04-24

**Authors:** Anna Pisacreta, Paolo Molinari, Lara Caldiroli, Margherita Di Naro, Francesco Pesce, Anna De Amici, Anna Regalia, Simona Verdesca, Silvia Malvica, Giuseppe Grandaliano, Giuseppe Castellano, Carlo Alfieri

**Affiliations:** 1 Department of Nephrology, Dialysis and Renal Transplantation, Fondazione IRCCS Ca’ Granda Ospedale Policlinico, 20122 Milan, Italylara.caldiroli@policlinico.mi.it (L.C.); margherita.dinaro@unimi.it (M.D.N.); silvia.malvica@policlinico.mi.it (S.M.);; 2 Department of Clinical Sciences and Community Health, University of Milan, 20122 Milan, Italy; 3 Division of Renal Medicine, Ospedale Isola Tiberina—Gemelli Isola, 00186 Rome, Italy; 4 Faculty of Medicine and Surgery, Università Cattolica del Sacro Cuore, 00168 Rome, Italy; 5 Nephrology Unit, Department of Medical and Surgical Sciences, Fondazione Policlinico Universitario A. Gemelli IRCCS, 00168 Rome, Italy

**Keywords:** sarcopenia, kidney transplantation, muscle wasting, sarcopenic obesity

## Abstract

Sarcopenia, defined as the progressive loss of skeletal muscle mass and strength, is increasingly recognized as a significant concern in patients with chronic kidney disease (CKD) and particularly in kidney transplant recipients (KTx-ps). This review explores the complex interplay of pathophysiological mechanisms, prevalence, and management strategies of sarcopenia in the context of kidney transplantation. CKD contributes to sarcopenia through systemic inflammation, malnutrition, uremic toxin accumulation, and metabolic imbalances, all of which persist or are exacerbated after transplantation due to immunosuppressive therapies especially corticosteroids. Notably, the post-transplant period may introduce additional risks, such as altered body composition and reduced physical activity, further aggravating muscle wasting. Sarcopenia affects approximately 26% of KTx-ps, leading to adverse outcomes including decreased quality of life, increased risk of infection, frailty, delayed recovery, and graft loss. The diagnosis remains challenging due to variability in assessment tools and a lack of standardized criteria. Management strategies must be multifactorial, including personalized nutritional support, targeted physical activity, and, where appropriate, pharmacological interventions. Early identification through imaging and functional testing is critical, especially in older patients and those with prolonged dialysis vintage. Emerging therapies, such as myostatin inhibitors, offer promise but require further validation. Additionally, early steroid withdrawal may mitigate muscle loss without compromising graft survival in selected patients. This review underscores the need for heightened awareness and standardized protocols to identify and manage sarcopenia in kidney transplantation, ultimately improving long-term outcomes and patient-centered care.

## 1. Introduction

Sarcopenia is a disorder characterized by the progressive and generalized loss of skeletal muscle strength and mass, associated with adverse outcomes including falls, disability, poor quality of life, and increased overall morbidity and mortality [1,2,3,4,5]. It can be classified as primary, related to ageing, or secondary, when it develops in the context of chronic diseases such as chronic kidney disease (CKD), type 2 diabetes mellitus (T2DM), long-term glucocorticoid exposure, and obesity [6,7].

**Figure 1:**
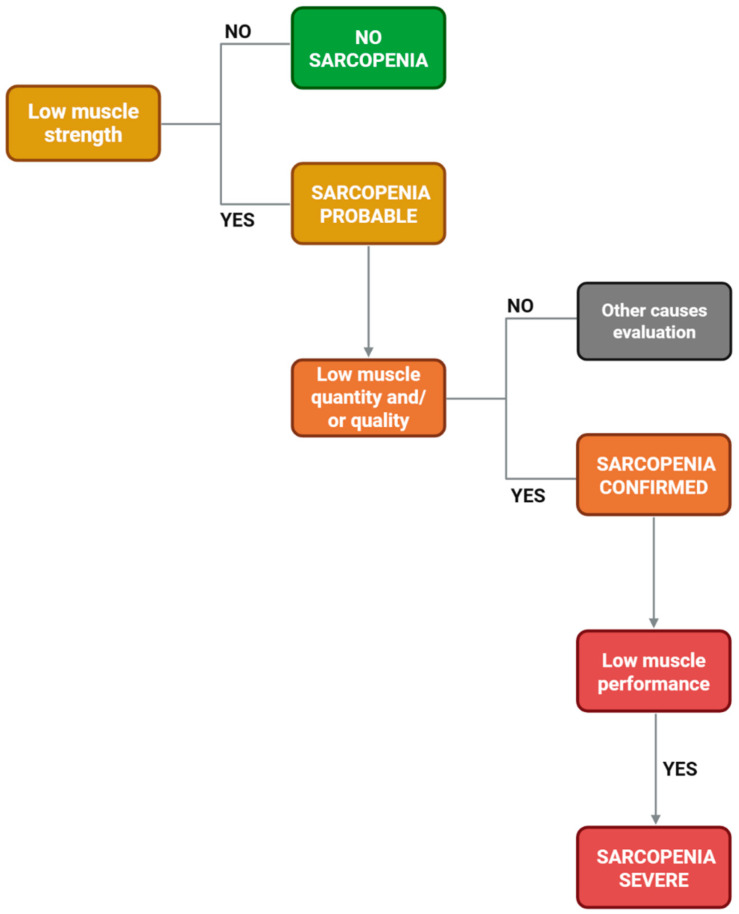
Diagnostic flowchart for sarcopenia according to the EWGSOP2 algorithm.

According to the European Working Group on Sarcopenia in Older People (EWGSOP2) definition, low muscle strength is the key initial criterion (probable sarcopenia). The diagnosis is confirmed when low muscle quantity and/or quality is also documented, and severity is defined by impaired physical performance (Figure 1) [1,8].

In CKD, sarcopenia is a frequent and clinically meaningful complication. Indeed, CKD promotes a “premature ageing” phenotype through systemic inflammation, malnutrition and uremic toxins accumulation. Furthermore, metabolic disturbances associated with CKD may disrupt cellular homeostasis and favour catabolic and senescence-related pathways [9,10,11]. Consequently, patients with CKD have a higher risk of sarcopenia than the general population, and its prevalence increases as kidney function declines [12,13]. Recent studies suggest that approximately 30–40% of patients with CKD across different stages exhibit sarcopenia, with higher rates among those receiving dialysis [14].

Kidney transplantation (KTx) improves survival and quality of life compared with dialysis and remains the preferred treatment for eligible patients with chronic kidney failure [15,16]. Nevertheless, sarcopenia may represent an under-recognized but clinically relevant problem in kidney transplant recipients (KTRs) [17]. Sarcopenia may coexist and partially overlap with frailty, reducing physiologic reserve and the ability to respond to perioperative and post-transplant stressors [18,19,20,21,22,23,24,25,26,27]. Several studies also suggest that sarcopenia may be associated with worse post-transplant outcomes and complications, including adverse graft-related outcomes in selected cohorts [28,29]. In summary, within the transplantation setting, two distinct forms of sarcopenia can be identified: pre-existing sarcopenia, present prior to transplantation, and post-transplant sarcopenia, which develops thereafter and whose prognostic significance has yet to be clearly established.

While pre-existing sarcopenia is a well-defined entity and a known burden, less has been evaluated regarding post-KTx sarcopenia. Post-transplant, the pooled prevalence of sarcopenia is approximately 26% (95% CI: 20–34%), though this varies considerably based on diagnostic criteria used, ranging from 7% using FNIH criteria to 17% using EWGSOP criteria and up to 50% using EWGSOP1 criteria [25].

One of the main challenges lies in the considerable heterogeneity among studies with regard to definitions of sarcopenia, assessment methodologies, and assessment timing. These inconsistencies contribute to wide variability in reported prevalence estimates and significantly limit comparability between cohorts. Furthermore, this methodological divergence hinders the translation of available evidence into standardized, evidence-based care pathways for transplants.

This review aims to summarize current evidence on the epidemiology, pathophysiology, and management of sarcopenia in kidney transplant recipients, with a focus on prevention and practical strategies that can be integrated into the transplant care continuum.

## 2. Sarcopenia in Kidney Transplantation

Kidney transplantation evolves across distinct clinical trajectory during which muscle health can change substantially from waitlisting to the early post-transplant period and long-term follow-up. Sarcopenia is therefore a dynamic condition in KTRs. A clinically relevant proportion of patients who are not sarcopenic before transplantation may develop sarcopenia shortly after surgery, consistent with the catabolic and deconditioning stress around surgery [30,31,32]. Early muscle wasting is influenced by baseline muscle reserves, dialysis vintage, reduced physical activity, and the intensity of immunosuppressive therapy, which is typically greatest soon after transplantation [7,10,11,33]. Corticosteroids, still included in many maintenance regimens, have well-established catabolic effects on skeletal muscle and may further amplify early declines in strength and muscle quantity and quality [34,35]. Across transplant cohorts, advanced age, diabetes mellitus, frailty, reduced physical activity, and impaired nutritional status are consistently reported as key correlates of adverse body composition phenotypes.

In this setting age plays a key role. Although the average age of patients with a kidney transplant is usually lower than that of non-transplanted CKD patients, the expansion of eligibility criteria for kidney transplantation is leading to a higher proportion of kidney transplant recipients being of advanced age. Age represents a significant risk factor for sarcopenia development in kidney transplant recipients. A systematic review and meta-analysis of 2535 kidney transplant recipients demonstrated that higher age was independently associated with increased sarcopenia risk (OR: 1.08, 95% CI: 1.05–1.10), indicating that each additional year of age confers approximately an 8% increase in sarcopenia likelihood [25].

Moreover, some of the risk factors present before KTx improve or resolve after KTx. Uremia-related factors play a major role in pre-transplant sarcopenia but are largely resolved after kidney transplantation. Accumulated uremic toxins impair muscle regeneration, promote protein degradation, and cause mitochondrial dysfunction, while pro-cachectic factors such as activin A further drive muscle wasting through disrupted kidney–muscle signaling. Metabolic acidosis and dialysis-related processes exacerbate catabolism, and multiple hormonal imbalances (e.g., insulin resistance, low testosterone, vitamin D deficiency) contribute to muscle loss. After transplantation, restoration of kidney function removes or improves many of these mechanisms, although some alterations, particularly mitochondrial dysfunction, may only partially recover.

Importantly, risk stratification should extend beyond muscle quantity. Sarcopenic obesity, characterized by reduced muscle strength in the setting of increased peri- and intramuscular adiposity, may be under-recognized. Indeed, conventional body-composition approaches can underestimate muscle impairment when excess fat masks low muscle mass and poor muscle function [31,34,36,37,38]. In addition, imaging-based markers of muscle quality, including intramuscular fat infiltration (myosteatosis), have been associated with an adverse cardiometabolic risk profile and, in selected studies, with worse post-transplant outcomes [11,32,35,39].

**Figure 2:**
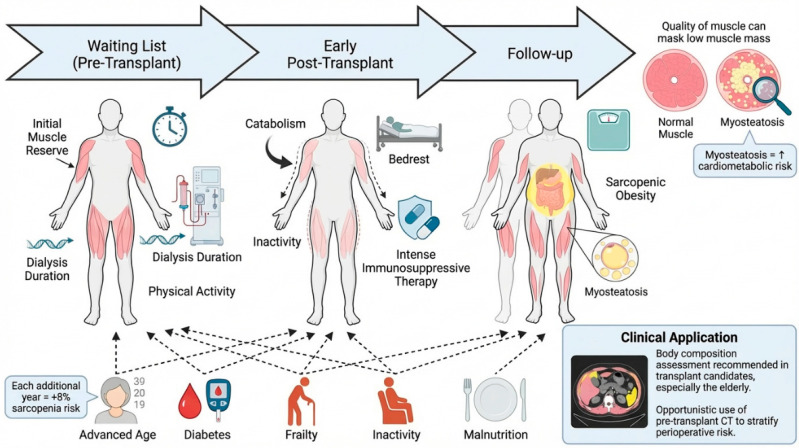
Sarcopenia development and risk factors in kidney transplantation.

These considerations extend to transplant candidates on the waiting list, particularly older patients [40]. When available, pretransplant CT obtained for surgical or vascular planning can enable opportunistic body-composition assessment that complements bedside functional testing and may refine perioperative risk stratification (Figure 2) [41,42].

### 2.1. Epidemiology

The prevalence of sarcopenia in kidney transplant recipients (KTRs) is clinically significant [27,28,29]. However, reported estimates vary widely across cohorts because definitions, measurement tools, and timing of assessment differ. Overall, recent data suggests a prevalence of approximately 26% among KTRs. However, prevalence estimates are higher when sarcopenia is defined solely by low muscle mass, and lower when diagnostic criteria require the concomitant presence of reduced muscle mass together with decreased muscle strength and/or impaired physical performance [25]. These considerations support interpreting prevalence estimates only in relation to the diagnostic construct applied in each study, such as BIA- or DXA-based low muscle mass versus combined criteria incorporating strength and physical performance.

Methodological issues are therefore central. Most consensus frameworks, including EWGSOP2, apply fixed, sex-specific cut-offs that were largely derived from older populations and are not routinely age-stratified. In transplant cohorts that often include younger and middle-aged recipients, this may contribute to under-recognition of clinically meaningful strength decline and can influence both prevalence estimates and risk stratification.

### 2.2. Diagnostic Assessment

Despite the existence of consensus definitions, sarcopenia assessment remains heterogeneous across studies and clinical settings. Muscle strength is most often measured with handgrip dynamometry, a low-cost and non-invasive tool suitable for routine practice, and values below established cut-offs correlate with clinically relevant weakness [1]. Lower-limb function can be assessed with the Short Physical Performance Battery (SPPB), which includes the chair-stand component and provides complementary information on mobility and functional reserve. For muscle quantity, dual-energy X-ray absorptiometry (DXA), computed tomography (CT), and magnetic resonance imaging (MRI) are reference standards, although their use may be limited by cost, availability, and logistics compared with bedside functional testing [43]. Bioimpedance analysis represents a practical alternative to estimate appendicular skeletal mass (ASM), reported as an absolute value or normalized to height (ASM/m^2^), and is suitable for repeated outpatient follow-up [43,44]. Muscle ultrasound is an increasingly adopted portable technique that can be integrated into routine evaluations by trained operators. It typically measures anterior thigh muscle thickness and echo intensity (often at the rectus femoris), with quadriceps thickness providing a useful surrogate of strength even in advanced wasting [44] (Table 1).

In transplant populations, the operational definition used becomes a key determinant of case identification and risk stratification. Current consensus definitions have predominantly been validated in geriatric populations and do not incorporate systematic age stratification. Consequently, they may be less applicable to transplant cohorts, potentially reducing diagnostic precision in this heterogeneous population. The ELSA-Brasil analysis provides a valuable reference-based framework by deriving age-stratified handgrip strength thresholds, thereby accounting for the physiological decline in muscle strength across adulthood and highlighting the limitations of fixed cut-offs, which may underestimate impairment in younger individuals. Collectively, these findings support an age-informed interpretation of HGS when screening for adverse body composition phenotypes in populations extending beyond the elderly [45] (Table 2).

A practical approach to transplant care can be taken step by step. First, screen for low muscle strength using either HGS or the chair-stand test. Secondly, if low muscle strength is detected, or if the patient is clinically high risk, confirm sarcopenia by documenting low muscle quantity or poor muscle quality using BIA or DXA. When available, use opportunistic CT- or ultrasound-based indices. Thirdly, determine the severity of the condition by measuring physical performance, for example, using the gait speed test or the SPPB test. Finally, interpret any discordant results in the clinical context. Low strength with preserved mass may still indicate clinically relevant impairment, particularly in obese patients. Low muscle mass without clear functional impairment may necessitate closer surveillance rather than immediate labelling. If imaging suggests reduced muscle density or increased fat infiltration, then myosteatosis and sarcopenic obesity should be considered [1,35,46,47,48,49].

Assessment should be conducted at meaningful time points along the transplant continuum. A baseline evaluation during waitlisting can guide prehabilitation. Reassessment in the early post-transplant period is reasonable, when catabolism and deconditioning are most prominent, and again at 3–6 months to capture recovery trajectories. Periodic follow-up—for example, annually—can detect later decline and emerging sarcopenic obesity. Additional assessments may be prompted by intercurrent events such as hospitalization, severe infection, rejection treatment, development of post-transplant diabetes, rapid weight change, or a noticeable decline in physical function [50,51,52,53].

**Figure 3:**
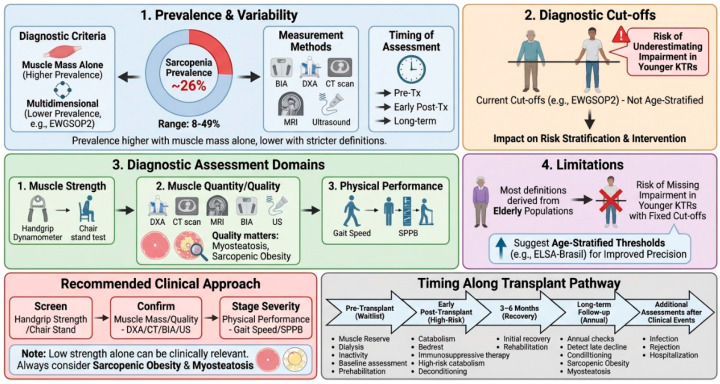
Epidemiology and assessment of sarcopenia in KTRs.

For research comparability, studies should clearly report the sarcopenia definition used, the tools and units applied for each domain, and the timing of assessment. Reporting should include one functional measure (e.g., HGS or chair-stand/SPPB) and one measure of muscle quantity or quality (BIA/DXA or opportunistic imaging) Figure 3 [1,48].

### 2.3. Pathophysiology

**Figure 4:**
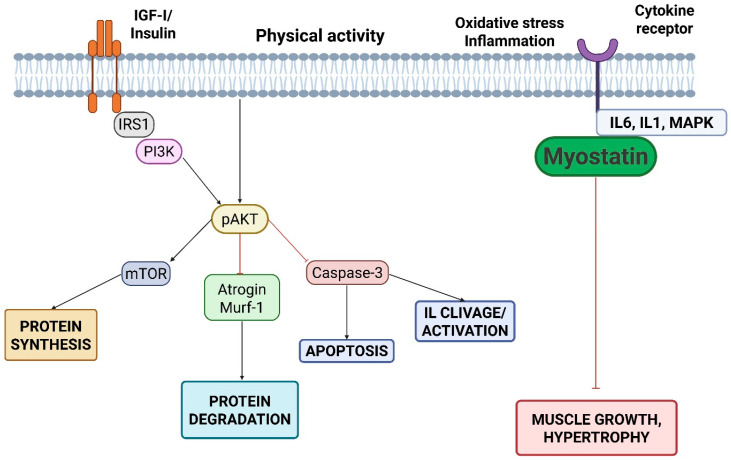
Imbalance between anabolic and catabolic pathways in sarcopenia. Note: IGF-I, insulin-like growth factor I; IRS1, insulin receptor substrate 1; PI3K, phosphoinositide 3-kinase; pAKT, phosphorylated protein kinase B (AKT); mTOR, mammalian target of rapamycin; MuRF-1, muscle RING finger 1; MAPK, mitogen-activated protein kinases; IL-6, interleukin 6; IL-1, interleukin 1.

Sarcopenia in CKD and KTRs results from a multifactorial imbalance between anabolic and catabolic signalling. This leads to a progressive loss of muscle strength, mass, and physical function [18,40]. It reflects a maladaptive interplay between intramuscular pathways and extrinsic stressors that chronically shifts the net muscle protein balance towards loss of force and decline in muscle quality. Several pathogenic mechanisms are initiated in advanced CKD and may persist after transplantation, whereas others are unique to transplantation or are amplified by the perioperative and post-transplant periods (Figure 4).

(a) **Intramuscular Factors**

**Figure 5:**
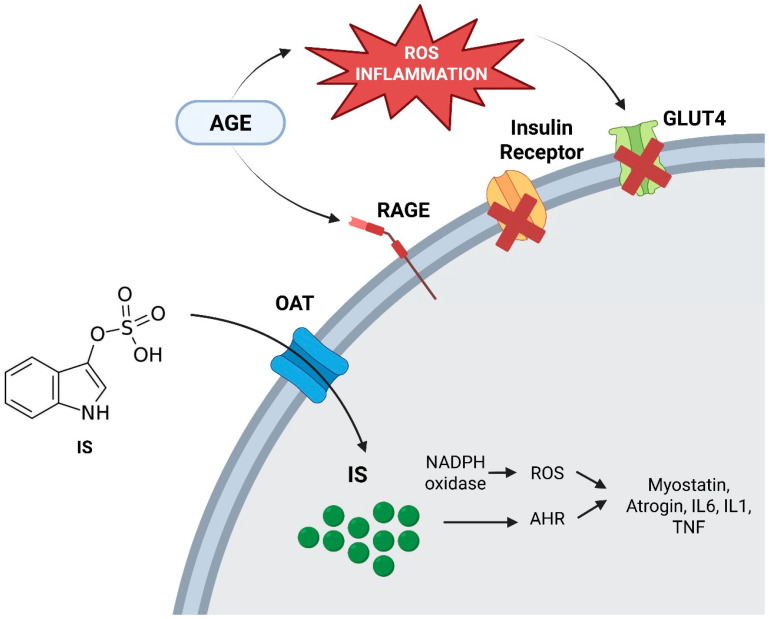
Mechanisms of oxidative stress and chronic inflammation in sarcopenia. [CMA5.1]. Note: AGE, advanced glycation end-products; ROS, reactive oxygen species; GLUT4, glucose transporter type 4; RAGE, receptor for advanced glycation end-products; IS, indoxyl sulfate; OAT, organic anion transporter; NADPH oxidase, nicotinamide adenine dinucleotide phosphate oxidase; AHR, aryl hydrocarbon receptor; IL-6, interleukin 6; IL-1, interleukin 1; TNF, tumor necrosis factor.

In healthy muscle, anabolic stimuli induced by physical exercise, dietary amino acids, hormones (e.g., insulin, growth hormone, testosterone), promote protein synthesis and muscle maintenance largely through PI3K/AKT/mTOR signalling. In CKD, this anabolic response is frequently attenuated (“anabolic resistance”). Concomitant insulin resistance and endocrine–metabolic derangements reduce anabolic signalling efficiency and favor proteolysis. In addition, uremic toxins can directly impair muscle homeostasis. Advanced glycation end products (AGEs) and protein-bound uremic toxins may promote oxidative stress and interfere with insulin signalling, and have been linked to increased myostatin activity, further shifting the balance toward catabolism and attenuated regeneration [40,41,42,54,55,56,57,58,59,60]. Oxidative stress also amplifies mitochondrial dysfunction and reduces muscle quality, which may partly explain why declines in strength and performance can occur even when muscle mass appears relatively preserved (Figure 5).

In KTRs, several transplant-specific factors may stimulate the expression of myostatin, thereby contributing to sarcopenia after transplantation. One such factor is chronic exposure to glucocorticoids, as these agents directly upregulate myostatin transcription and promote muscle proteolysis [61,62]. Post-transplant metabolic disturbances, including insulin resistance and new-onset diabetes, also impair insulin/IGF-1 signalling and are associated with increased myostatin activity [63]. Reduced physical activity and mechanical unloading, particularly in the early post-transplant phase, further enhanced myostatin expression due to the loss of contraction-mediated suppression [64]. Interference with muscle growth signalling by calcineurin inhibitors may also indirectly favour myostatin upregulation [65]. Finally, persistent low-grade inflammation, despite improved renal function, can contribute to myostatin activation through cytokine-mediated pathways [66]. Taken together, these mechanisms emphasise distinct, transplant-specific myostatin regulation, which differs from the uremia-driven pathways predominantly observed in CKD.

(b) **External Factors**

Several systemic and clinical factors further amplify muscle loss in CKD and KTRs. Chronic inflammation is a hallmark of CKD and variably persistent after transplantation, characterised by prolonged exposure to cytokines such as TNF-α, IL-6, and IL-2 [67,68]. These mediators activate muscle-degradative pathways, increase intracellular oxidative stress, and may stimulate autocrine myostatin signalling, thereby accelerating muscle wasting [67,68,69].

Malnutrition and reduced protein intake represent a second major axis. Nutritional deficits may occur in advanced CKD and can be reinforced by dietary restrictions, reduce amino acid availability and limit muscle repair and growth [69]. Notably, poor nutritional status before transplantation has been associated with adverse post-transplant outcomes, including delayed graft function and a higher risk of infection, highlighting the clinical relevance of this axis in the transplant setting [7,20]. Metabolic acidosis, when present and insufficiently corrected, provides an additional systemic pro-catabolic stimulus and contributes to loss of lean body mass.

After transplantation, several external drivers become transplant-unique or are amplified. The perioperative phase is characterized by acute inflammation, reduced oral intake, and immobilization, and can precipitate rapid catabolism and deconditioning, particularly in patients with limited pre-transplant muscle reserves and prolonged dialysis vintage [11,32]. Immunosuppression is a key contributor. Corticosteroids inhibit muscle protein synthesis and enhance protein breakdown and may therefore amplify early declines in muscle mass and strength [70]. Calcineurin inhibitors and the broader post-transplant metabolic environment may further influence muscle health indirectly through effects on glucose metabolism and cardiometabolic risk, including post-transplant diabetes mellitus (PTDM). PTDM, weight gain, and shifts in fat distribution can promote sarcopenic obesity and worsening muscle quality, including intramuscular fat infiltration. Intercurrent events common after transplantation, such as infections, rejection episodes and their treatment, and hospitalizations, increase inactivity and catabolic exposure and can delay functional recovery [56]. Finally, hypophosphatemia is frequent early after KTx and may impair muscle energy metabolism, representing an additional contributor to post-transplant weakness and wasting in susceptible patients [18].

Overall, sarcopenia in KTx-ps results from the interaction of intramuscular anabolic resistance and toxin/oxidative-stress pathways with systemic inflammation, malnutrition, inactivity, and transplant-specific stressors. This framework supports targeted strategies that address modifiable external drivers (nutrition, activity, acid–base status, management of PTDM and weight gain, prevention of deconditioning during hospitalizations) while minimizing avoidable catabolic exposures when clinically appropriate (e.g., steroid burden within risk-adapted immunosuppression protocols), providing a mechanistic bridge to the management section.

## 3. Management Strategies for Sarcopenia

The management of sarcopenia in CKD and kidney transplant recipients requires a comprehensive approach centered on early identification and proactive interventions, including optimizing of nutrition and promotion of physical activity both before and after kidney transplantation.

As stated earlier, in kidney transplantation, sarcopenia should be evaluated with age-specific considerations for diagnosis, treatment, and expected response to interventions. The transplant population is highly heterogeneous, ranging from younger patients with end-stage kidney disease to older recipients with multiple comorbidities and varying physiological reserves. Applying a single age cut-off may therefore fail to capture clinically meaningful differences in muscle loss, functional decline, and recovery potential. Adopting differentiated age thresholds and tailored assessment strategies could improve the identification and management of sarcopenia, ultimately allowing more personalized therapeutic approaches and better post-transplant outcomes.

Adequate amino acid intake, particularly from high-quality protein sources, is essential to support muscle protein synthesis [71]. Low-protein diets, often recommended during conservative CKD management, should be prescribed only when clearly indicated and should be preceded by a careful assessment of the patient’s habitual protein intake [72]. Given the heterogeneity of patients and clinical trajectories, individualized strategies with case-by-case risk–benefit evaluations are required to achieve optimal outcomes [73]. Amino acid supplementation, including branched-chain amino acids (BCAAs), has shown potential to reduce muscle wasting and support muscle protein synthesis in CKD and transplant populations [71].

Physical activity, especially combined aerobic and resistance training, confers multiple benefits in CKD and KTx. Primarily, it have favorable effects on inflammation, oxidative stress, immune response, and metabolism, as well as improvements in muscle mass, strength, functional capacity, and quality of life [74,75,76,77,78]. Several studies suggest that physical exercise significantly mitigates the clinical impact of sarcopenia by improving strength, mobility, and balance and by reducing cardiovascular risk, although its direct effect on kidney function remains uncertain [79,80,81].

In KTx-ps, both pre- and post-operative exercise are relevant. Pre-transplant “prehabilitation” programs may improve physical condition and potentially reduce perioperative risk through resistance and aerobic training and flexibility exercises. Post-transplant rehabilitation should be encouraged to optimize recovery, limit muscle loss, prevent complications, and restore physical function [82,83]. Regular exercise may also help control post-transplant comorbidities such as hypertension and post-transplant diabetes mellitus, while improving patient-reported outcomes [77,82]. Tailored programs have been associated with reduced fatigue and improved endurance and mobility during recovery [84,85,86]. Emerging data suggest a possible relationship between physical activity and therapeutic adherence in transplant recipients [87]. Overall, exercise prescriptions should be individualized according to comorbidities, baseline fitness, and post-operative limitations to maximize benefit and reduce injury risk. This individualized approach is key to ensuring long-term success in rehabilitation and improving the overall well-being of kidney transplant recipients.

Given the catabolic effects of corticosteroids, optimization of immunosuppressive therapy represents another potential strategy to reduce post-KTx sarcopenia. While corticosteroids are important for preventing organ rejection, prolonged exposure contributes to muscle wasting [30]. Early steroid reduction or withdrawal, when clinically feasible, may mitigate steroid-related complications, including steroid-induced diabetes and sarcopenia, without worsening transplant outcomes in selected recipients. In particular, several studies report that early steroid withdrawal does not increase long-term graft failure or mortality in patients with low-to-moderate immunological risk receiving tacrolimus- and mycophenolate mofetil-based regimens [88]. However, based on the available literature here, there is no direct published evidence demonstrating that steroid withdrawal mitigates sarcopenia in kidney transplant recipients. Existing studies largely focus on cardiovascular risk, metabolic parameters, and bone health; sarcopenia outcomes have not yet been formally linked to steroid withdrawal in this population. So, the evidence remains not fully conclusive, largely due to limited long-term prospective data, and early steroid withdrawal is generally not considered safe in re-transplant recipients, underscoring the need for individualized risk assessment [89,90].

In summary, while early steroid withdrawal appears to be an effective and safe strategy for many kidney transplant recipients, further research is needed. It is crucial to gather more data through long-term prospective studies to fully evaluate the benefits and potential risks, enabling a more tailored approach to treatment that minimizes complications, such as sarcopenia, without compromising graft survival and the patient’s quality of life.

Finally, myostatin inhibitors, such as antibodies, peptides, and soluble receptors, represent a promising and innovative therapeutic approach for enhancing muscle growth in CKD patients and KTx-ps with sarcopenia [91,92]. Myostatin is a key negative regulator of muscle growth, and its inhibition increase muscle mass in preclinical models [93]. Some data also suggest anti-myostatin strategies may reduce circulating inflammatory cytokines, potentially addressing a component of CKD-related systemic inflammation [91].

In a chronic kidney disease model, the administration of an anti-myostatin peptide in mice (via subcutaneous injection over approximately four weeks) resulted in a significant increase in muscle mass (approximately 10%) and body weight. This was accompanied by reduced protein degradation and enhanced insulin-like growth factor 1 (IGF-1) signalling [90]. Similarly, antisense oligonucleotides targeting myostatin (25–50 mg/kg for eight weeks) have been shown to improve muscle mass, fibre size and functional performance in CKD models by downregulating atrogenes such as MuRF-1 and atrogin-1 [91]. These findings suggest that myostatin blockade can reverse the key pathways involved in muscle atrophy. However, translation into clinical practice has been limited. Several myostatin/activin pathway inhibitors have been evaluated in phase II/III trials. Bimagrumab (BYM338), an activin type II receptor antagonist, increased lean body mass and reduced fat mass in randomised studies; however, effects on muscle strength and physical performance were modest or inconsistent [94]. Similarly, landogrozumab (LY2495655) increased appendicular lean mass in older adults with sarcopenia, but there were no consistent functional benefits [95]. Earlier agents such as stamulumab (MYO-029) demonstrated favourable safety profiles but limited efficacy [96].

Nonetheless, safety requires carefully evaluated, as myostatin shares structural similarities with other growth factors in the TGF-β family, meaning broad inhibition could potentially lead to undesirable side effects, such as vascular or bone damage [93]. Therefore, further research is needed to clarify efficacy and safety of myostatin inhibitors in clinical populations. Importantly, myostatin-targeted therapies should be viewed as complementary rather than alternatives to nutrition and exercise, which remain the foundation of sarcopenia management.

## 4. Conclusions

Sarcopenia is a complex, multifactorial condition that significantly impacts patients with CKD and kidney transplant recipients. Due to heterogeneous clinical tools are used for its assessment, the true prevalence of sarcopenia remains unclear both among candidates on the transplant waiting list and after kidney transplantation. Therefore, recognizing the heterogeneity of kidney transplant recipients and adopting age-specific cut-offs for sarcopenia may enable more accurate diagnosis and more personalized management strategies.

In CKD, particularly in advanced stages and during dialysis treatment, sarcopenia is primarily driven by the uremic environment, which promotes a catabolic state through inflammation, metabolic acidosis, oxidative stress, hormonal imbalances and protein-energy wasting. This leads to reduced muscle synthesis and increased proteolysis [97,98]. Kidney transplantation only partially reverses these mechanisms. Although uremia and inflammation improve, sarcopenia often persists or develops de novo. In KTRs, sarcopenia is largely influenced by transplant-specific factors, including immunosuppressive therapy (especially corticosteroids), insulin resistance, mitochondrial dysfunction, reduced physical activity and incomplete renal recovery [65,99]. Additionally, sarcopenic obesity is more prevalent than the protein-energy wasting phenotype typically observed in CKD [99].

This variability has likely contributed to under-recognition of the problem, limiting a thorough investigation of its determinants, which appear to be partly shared with mechanisms observed in the broader CKD population and partly transplant-specific (e.g., medication exposure and chronic inflammation). Consequently, corrective and preventive approaches have not been consistently developed or implemented.

A multifaceted strategy combining dietary interventions, physical exercise, and pharmacological support is essential to prevent and manage sarcopenia, with the aim of improving outcomes and quality of life in this vulnerable population.

**Figure 6:**
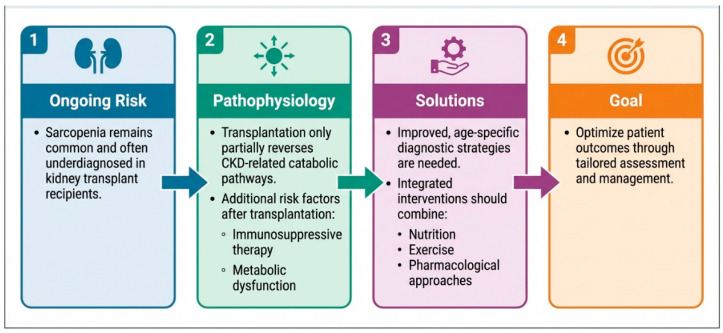
Sarcopenia in Kidney transplant recipients: persistent challenge and solutions.

Future research should prioritize shared, standardized assessment tools to better define prevalence and severity in kidney transplant recipients. In parallel, studies should clarify the factors driving onset and progression and evaluate targeted interventions, including lifestyle modification programs and both immunosuppressive and non-immunosuppressive therapeutic strategies (Figure 6).

## Figures and Tables

**Table 1:** EWGSOP2 diagnostic criteria and commonly used cut-offs for sarcopenia.

Measurement Tools	EWGSOP2-Based Cut-Offs
HGS	Men: <27 kg; Women: <16 kg
DXA	Men: <7.26 kg/m^2^; Women: <5.45 kg/m^2^
BIA	Men: <10 kg; Women: <7 kg
CT/MRI	<10th percentile of muscle volume
SPPB	<9 points

Note: HGS: Hand Grip Strength, DXA: Dual-Energy X-ray Absorptiometry, BIA: Bioelectrical Impedance Analysis, CT/MRI: Computed Tomography/Magnetic Resonance Imaging, SPPB: Short Physical Performance Battery.

**Table 2:** Handgrip strength (HGS) cut-offs: fixed EWGSOP2 thresholds versus age-stratified reference values from ELSA-Brasil.

Measurement Tools	EWGSOP2-Based Cut-Offs	Reference Age-Stratified Cut-Offs
HGS	Men: <27 kg; Women: <16 kg	Men (kg): 38–44 y ≤ 42
		45–54 y ≤ 41
		55–64 y ≤ 38
		65–79 y ≤ 36
		Women (kg): 38–44 y ≤ 26
		45–54 y ≤ 23
		55–64 y ≤ 23
		65–79 y ≤ 21

Note: HGS: Hand Grip Strength; EWGSOP2: European Working Group on Sarcopenia in Older People 2.

## Data Availability

No new data were created or analyzed in this study.

## References

[1] Cruz-Jentoft A.J., Bahat G., Bauer J., Boirie Y., Bruyère O., Cederholm T., Cooper C., Landi F., Rolland Y., Sayer A.A. (2019). Sarcopenia: Revised European consensus on definition and diagnosis. Age Ageing.

[2] Rizzoli R., Reginster J.Y., Arnal J.F., Bautmans I., Beaudart C., Bischoff-Ferrari H., Biver E., Boonen S., Brandi M.-L., Chines A. (2013). Quality of life in sarcopenia and frailty. Calcif. Tissue Int..

[3] Visser M., Schaap L.A. (2011). Consequences of sarcopenia. Clin. Geriatr. Med..

[4] Lang T., Streeper T., Cawthon P., Baldwin K., Taaffe D.R., Harris T.B. (2010). Sarcopenia: Etiology, clinical consequences, intervention, and assessment. Osteoporos. Int..

[5] Landi F., Liperoti R., Russo A., Giovannini S., Tosato M., Capoluongo E., Bernabei R., Onder G. (2012). Sarcopenia as a risk factor for falls in elderly individuals: Results from the ilSIRENTE study. Clin. Nutr..

[6] Yanishi M., Kimura Y., Tsukaguchi H., Koito Y., Taniguchi H., Mishima T., Fukushima Y., Sugi M., Kinoshita H., Matsuda T. (2017). Development of Sarcopenia in Kidney Transplant Recipients. Transplant. Proc..

[7] Fielding R.A., Vellas B., Evans W.J., Bhasin S., Morley J.E., Newman A.B., van Kan G.A., Andrieu S., Bauer J., Breuille D. (2011). Sarcopenia: An undiagnosed condition in older adults. Current consensus definition: Prevalence, etiology, and consequences. International working group on sarcopenia. J. Am. Med. Dir. Assoc..

[8] Cruz-Jentoft A.J., Baeyens J.P., Bauer J.M., Boirie Y., Cederholm T., Landi F., Martin F.C., Michel J.-P., Rolland Y., Schneider S.M. (2010). Sarcopenia: European consensus on definition and diagnosis: Report of the European Working Group on Sarcopenia in Older People. Age Ageing.

[9] Sabatino A., Regolisti G., Karupaiah T., Sahathevan S., Sadu Singh B.K., Khor B., Salhab N., Karavetian M., Cupisti A., Fiaccadori E. (2017). Protein-energy wasting and nutritional supplementation in patients with end-stage renal disease on hemodialysis. Clin. Nutr..

[10] Watanabe H., Enoki Y., Maruyama T. (2019). Sarcopenia in Chronic Kidney Disease: Factors, Mechanisms, and Therapeutic Interventions. Biol. Pharm. Bull..

[11] Kim J.K., Choi S.R., Choi M.J., Kim S.G., Lee Y.K., Noh J.W., Kim H.J., Song Y.R. (2014). Prevalence of and factors associated with sarcopenia in elderly patients with end-stage renal disease. Clin. Nutr..

[12] Ribeiro H.S., Neri S.G.R., Oliveira J.S., Bennett P.N., Viana J.L., Lima R.M. (2022). Association between sarcopenia and clinical outcomes in chronic kidney disease patients: A systematic review and meta-analysis. Clin. Nutr..

[13] Chatzipetrou V., Bégin M.J., Hars M., Trombetti A. (2022). Sarcopenia in Chronic Kidney Disease: A Scoping Review of Prevalence, Risk Factors, Association with Outcomes, and Treatment. Calcif. Tissue Int..

[14] Duarte M.P., Almeida L.S., Neri S.G.R., Oliveira J.S., Wilkinson T.J., Ribeiro H.S., Lima R.M. (2024). Prevalence of sarcopenia in patients with chronic kidney disease: A global systematic review and meta-analysis. J. Cachexia Sarcopenia Muscle.

[15] Tonelli M., Wiebe N., Knoll G., Bello A., Browne S., Jadhav D., Klarenbach S., Gill J. (2011). Systematic review: Kidney transplantation compared with dialysis in clinically relevant outcomes. Am. J. Transplant..

[16] Port F.K., Wolfe R.A., Mauger E.A., Berling D.P., Jiang K. (1993). Comparison of survival probabilities for dialysis patients vs. cadaveric renal transplant recipients. JAMA.

[17] Chadban S.J., Ahn C., Axelrod D.A., Foster B.J., Kasiske B.L., Kher V.M., Kumar D.M., Oberbauer R., Pascual J., Pilmore H.L. (2020). KDIGO Clinical Practice Guideline on the Evaluation and Management of Candidates for Kidney Transplantation. Transplantation.

[18] Gandolfini I., Regolisti G., Bazzocchi A., Maggiore U., Palmisano A., Piotti G., Fiaccadori E., Sabatino A. (2019). Frailty and Sarcopenia in Older Patients Receiving Kidney Transplantation. Front. Nutr..

[19] Harada H., Nakamura M., Hotta K., Iwami D., Seki T., Togashi M., Hirano T., Miyazaki C. (2012). Percentages of water, muscle, and bone decrease and lipid increases in early period after successful kidney transplantation: A body composition analysis. Transplant. Proc..

[20] Deliège P.G., Braconnier A., Chaix F., Renard Y., Petrache A., Guyot-Colosio C., Kazes I., Mokri L., Barbe C., Rieu P. (2021). Skeletal Muscle Index as a Prognostic Marker for Kidney Transplantation in Older Patients. J. Ren. Nutr..

[21] Alfieri C., Malvica S., Cesari M., Vettoretti S., Benedetti M., Cicero E., Miglio R., Caldiroli L., Perna A., Cervesato A. (2022). Frailty in kidney transplantation: A review on its evaluation, variation and long-term impact. Clin. Kidney J..

[22] Streja E., Molnar M.Z., Kovesdy C.P., Bunnapradist S., Jing J., Nissenson A.R., Mucsi I., Danovitch G.M., Kalantar-Zadeh K. (2011). Associations of pretransplant weight and muscle mass with mortality in renal transplant recipients. Clin. J. Am. Soc. Nephrol..

[23] Garonzik-Wang J.M., Govindan P., Grinnan J.W., Liu M., Ali H.M., Chakraborty A., Jain V., Ros R.L., James N.T., Kucirka L.M. (2012). Frailty and delayed graft function in kidney transplant recipients. Arch. Surg..

[24] McAdams-DeMarco M.A., Law A., King E., Orandi B., Salter M., Gupta N., Chow E., Alachkar N., Desai N., Varadhan R. (2015). Frailty and mortality in kidney transplant recipients. Am. J. Transplant..

[25] Zhang J.Z., Shi W., Zou M., Zeng Q.S., Feng Y., Luo Z., Gan H. (2023). Diagnosis, prevalence, and outcomes of sarcopenia in kidney transplantation recipients: A systematic review and meta-analysis. J. Cachexia Sarcopenia Muscle.

[26] Kosoku A., Iwai T., Kabei K., Nishide S., Machida Y., Ishihara T., Uchida J. (2024). Sarcopenia as a predictor of mortality in kidney transplant recipients: A 5-year prospective cohort study with propensity score matching. Int. J. Urol..

[27] Karakizlis H., Trudel N., Brose A., Reinisch A., Reichert M., Hecker A., Bender F., Askevold I., Rainer L., Weimer R. (2023). Sarcopenia of kidney transplant recipients as a predictive marker for reduced graft function and graft survival after kidney transplantation. Langenbecks Arch. Surg..

[28] Ozcan S.G., Sonmez O., Atli Z., Karaca C., Alagoz S., Akman Z., Koroglu A.E., Pekmezci S., Trabulus S., Seyahi N. (2024). Sarcopenia, an overlooked diagnosis in kidney transplant recipients. Clin. Nephrol..

[29] Martins C.A., Franga A.K.T.D.C., Dias R.S.C., Costa R.C.O., Lemos A.P.L., dos Santos A.M., Hortegal E.V., Brito D.J.d.A. (2020). Prevalence of sarcopenia in kidney transplants and their association with determinant factors of muscle homeostasis. Rev. Assoc. Med. Bras..

[30] Nanmoku K., Kawabata N., Kinoshita Y., Shinzato T., Kubo T., Shimizu T., Yagisawa T. (2020). Deterioration of presarcopenia and its risk factors following kidney transplantation. Clin. Exp. Nephrol..

[31] Mazzola A., Brustia R., Magro B., Atif M., Ouali N., Tourret J., Barrou B., Scatton O., Conti F. (2021). Impact of sarcopenia on clinical outcomes of patients undergoing simultaneous liver and kidney transplantation: A cohort study. Clin. Res. Hepatol. Gastroenterol..

[32] Bellafronte N.T., Sizoto G.R., Vega-Piris L., Chiarello P.G., Cuadrado G.B. (2020). Bed-side measures for diagnosis of low muscle mass, sarcopenia, obesity, and sarcopenic obesity in patients with chronic kidney disease under non-dialysis-dependent, dialysis dependent and kidney transplant therapy. PLoS ONE.

[33] Takamoto D., Kawahara T., Mochizuki T., Makiyama K., Teranishi J., Uemura H. (2018). A Longer History of Hemodialysis Can Lead to Sarcopenia in Renal Transplantation Patients. Transplant. Proc..

[34] van den Ham E.C., Kooman J.P., Christiaans M.H., Leunissen K.M., van Hooff J.P. (2000). Posttransplantation weight gain is predominantly due to an increase in body fat mass. Transplantation.

[35] Morel A., Ouamri Y., Canoui-Poitrine F., Mulé S., Champy C.M., Ingels A., Audard V., Luciani A., Grimbert P., Matignon M. (2022). Myosteatosis as an independent risk factor for mortality after kidney allograft transplantation: A retrospective cohort study. J. Cachexia Sarcopenia Muscle.

[36] Dienemann T., Ziolkowski S.L., Bender S., Goral S., Long J., Baker J.F., Shults J., Zemel B.S., Reese P.P., Wilson F.P. (2021). Changes in Body Composition, Muscle Strength, and Fat Distribution Following Kidney Transplantation. Am. J. Kidney Dis..

[37] Zamboni M., Rubele S., Rossi A.P. (2019). Sarcopenia and obesity. Curr. Opin. Clin. Nutr. Metab. Care.

[38] Kosoku A., Iwai T., Kabei K., Nishide S., Machida Y., Uchida J. (2023). Frailty and sarcopenia in older kidney transplant recipients: A cross-sectional study. Eur. Geriatr. Med..

[39] Li Y., Chen T., Zhang Z., Fan Y., Lin T., Chen J., Song T. (2024). Sarcopenic obesity is associated with adverse outcomes after kidney transplantation: A retrospective cohort study. Int. Urol. Nephrol..

[40] Ponticelli C., Podestà M.A., Graziani G. (2014). Renal transplantation in elderly patients. How to select the candidates to the waiting list?. Transplant. Rev..

[41] Quint E.E., Liu Y., Shafaat O., Ghildayal N., Crosby H., Kamireddy A., Pol R.A., Orandi B.J., Segev D.L., Weiss C.R. (2024). Abdominal computed tomography measurements of body composition and waitlist mortality in kidney transplant candidates. Am. J. Transplant..

[42] Alavi D.H., Henriksen H.B., Lauritzen P.M., Kværner A.S., Sakinis T., Langleite T.M., Henriksen C., Bøhn S.K., Paur I., Wiedswang G. (2021). Quantification of adipose tissues by Dual-Energy X-Ray Absorptiometry and Computed Tomography in colorectal cancer patients. Clin. Nutr. ESPEN.

[43] Han A., Bokshan S.L., Marcaccio S.E., DePasse J.M., Daniels A.H. (2018). Diagnostic Criteria and Clinical Outcomes in Sarcopenia Research: A Literature Review. J. Clin. Med..

[44] Fu H., Wang L., Zhang W., Lu J., Yang M. (2023). Diagnostic test accuracy of ultrasound for sarcopenia diagnosis: A systematic review and meta-analysis. J. Cachexia Sarcopenia Muscle.

[45] Santos C.A., Maia H.F., Pitanga F.J.G., de Almeida M.D.C.C., da Fonseca M.J.M., de Aquino E.M.L., Cardoso L.d.O., Griep R.H., Barreto S.M., Suemoto C.K. (2025). Hand Grip Strength Cut-Off Points as a Discriminator of Sarcopenia and Sarcopenic Obesity: Results from the ELSA-Brasil Cohort. J. Cachexia Sarcopenia Muscle.

[46] Coban H., Barutcu Atas D., Tugcu M., Kursun M., Cimsit C., Asicioglu E., Arikan H., Tuglular S., Velioglu A. (2024). Computed Tomography-Assessed Sarcopenia Predicts Mortality in Kidney Transplant Candidates. Exp. Clin. Transplant..

[47] Boutin R.D., Lenchik L. (2020). Value-Added Opportunistic CT: Insights into Osteoporosis and Sarcopenia. AJR Am. J. Roentgenol..

[48] Zanker J., Sim M., Anderson K., Balogun S., Brennan-Olsen S.L., Dent E., Duque G., Girgis C.M., Grossmann M., Hayes A. (2023). Consensus guidelines for sarcopenia prevention, diagnosis and management in Australia and New Zealand. J. Cachexia Sarcopenia Muscle.

[49] Chen L.K., Woo J., Assantachai P., Auyeung T.W., Chou M.Y., Iijima K., Jang H.C., Kang L., Kim M., Kim S. (2020). Asian Working Group for Sarcopenia: 2019 Consensus Update on Sarcopenia Diagnosis and Treatment. J. Am. Med. Dir. Assoc..

[50] Quint E.E., Haanstra A.J., van der Veen Y., Maring H., Berger S.P., PreCareTx Investigators (2023). PREhabilitation of CAndidates for REnal Transplantation (PreCareTx) study: Protocol for a hybrid type I, mixed method, randomised controlled trial. BMJ Open.

[51] Alrashidi F.S., AlDawsari M.A., Negm H.A., AlAtmi A.A. (2026). Frailty Screening and Prehabilitation Before Kidney Transplant Listing: A Practical Narrative Review. Cureus.

[52] Pérez-Sáez M.J., Muñoz-Redondo E., Morgado-Pérez A., Delcros-Forestier L., Bach A., FRAILMar Study Group (2025). Exercise-Based Prehabilitation for Kidney Transplant Candidates: The FRAILMar Randomized Controlled Trial. Am. J. Kidney Dis..

[53] Ferrer-López E., López-Blasco R., Rubio-Castañeda F.J., Cantín-Lahoz V., Aguilón-Leiva J.J., García-Magán M., Navas-Ferrer C., Blázquez-Ornat I., Fernández-Rodrigo M.T., Antón-Solanas I. (2025). Changes in Body Composition Compartments After Kidney Transplantation: A One-Year Prospective Study. J. Clin. Med..

[54] Verzola D., Picciotto D., Saio M., Aimasso F., Bruzzone F., Sukkar S.G., Massarino F., Esposito P., Viazzi F., Garibotto G. (2020). Low Protein Diets and Plant-Based Low Protein Diets: Do They Meet Protein Requirements of Patients with Chronic Kidney Disease?. Nutrients.

[55] Siew E.D., Ikizler T.A. (2010). Insulin resistance and protein energy metabolism in patients with advanced chronic kidney disease. Semin. Dial..

[56] Esposito P., Picciotto D., Battaglia Y., Costigliolo F., Viazzi F., Verzola D. (2022). Myostatin: Basic biology to clinical application. Adv. Clin. Chem..

[57] Bunn R.C., Adatorwovor R., Smith R.R., Ray P.D., Fields S.E., Keeble A.R., Fry C.S., Uppuganti S., Nyman J.S., Fowlkes J.L. (2023). Pharmacologic Inhibition of Myostatin with a Myostatin Antibody Improves the Skeletal Muscle and Bone Phenotype of Male Insulin-Deficient Diabetic Mice. J. Bone Miner. Res. Plus.

[58] Andres-Hernando A., Cicerchi C., Garcia G.E., Orlicky D.J., Stenvinkel P., Johnson R.J., Lanaspa M.A. (2023). Phosphate depletion in insulin-insensitive skeletal muscle drives AMPD activation and sarcopenia in chronic kidney disease. iScience.

[59] Dozio E., Vettoretti S., Lungarella G., Messa P., Corsi Romanelli M.M. (2021). Sarcopenia in Chronic Kidney Disease: Focus on Advanced Glycation End Products as Mediators and Markers of Oxidative Stress. Biomedicines.

[60] Enoki Y., Watanabe H., Arake R., Sugimoto R., Imafuku T., Tominaga Y., Ishima Y., Kotani S., Nakajima M., Tanaka M. (2016). Indoxyl sulfate potentiates skeletal muscle atrophy by inducing the oxidative stress-mediated expression of myostatin and atrogin-1. Sci. Rep..

[61] Ma K., Mallidis C., Bhasin S., Mahabadi V., Artaza J., Gonzalez-Cadavid N., Arias J., Salehian B. (2003). Glucocorticoid-induced skeletal muscle atrophy is associated with upregulation of myostatin gene expression. Am. J. Physiol. Endocrinol. Metab..

[62] Schakman O., Kalista S., Barbé C., Loumaye A., Thissen J.P. (2013). Glucocorticoid-induced skeletal muscle atrophy. Int. J. Biochem. Cell Biol..

[63] Cochet C., Belloni G., Buondonno I., Chiara F., D’Amelio P. (2023). The Role of Nutrition in the Treatment of Sarcopenia in Old Patients: From Restoration of Mitochondrial Activity to Improvement of Muscle Performance, a Systematic Review. Nutrients.

[64] Allen D.L., Hittel D.S., McPherron A.C. (2011). Expression and function of myostatin in obesity, diabetes, and exercise adaptation. Med. Sci. Sports Exerc..

[65] Stenvinkel P., Carrero J.J., von Walden F., Ikizler T.A., Nader G.A. (2016). Muscle wasting in end-stage renal disease promulgates premature death: Established, emerging and potential novel treatment strategies. Nephrol. Dial. Transplant..

[66] Zhang L., Pan J., Dong Y., Tweardy D.J., Dong Y., Garibotto G., Mitch W.E. (2013). Stat3 activation links a C/EBPδ to myostatin pathway to stimulate loss of muscle mass. Cell Metab..

[67] Esposito P., Verzola D., Saio M., Picciotto D., Frascio M., Laudon A., Zanetti V., Brunori G., Garibotto G., Viazzi F. (2023). The Contribution of Muscle Innate Immunity to Uremic Cachexia. Nutrients.

[68] Carrero J.J., Chmielewski M., Axelsson J., Snaedal S., Heimburger O., Bárány P., Suliman M.E., Lindholm B., Stenvinkel P., Qureshi A.R. (2008). Muscle atrophy, inflammation and clinical outcome in incident and prevalent dialysis patients. Clin. Nutr..

[69] Bataille S., Chauveau P., Fouque D., Aparicio M., Koppe L. (2021). Myostatin and muscle atrophy during chronic kidney disease. Nephrol. Dial. Transplant..

[70] Hasselgren P.O., Alamdari N., Aversa Z., Gonnella P., Smith I.J., Tizio S. (2010). Corticosteroids and muscle wasting: Role of transcription factors, nuclear cofactors, and hyperacetylation. Curr. Opin. Clin. Nutr. Metab. Care.

[71] Hahn D., Hodson E.M., Fouque D. (2018). Low protein diets for non-diabetic adults with chronic kidney disease. Cochrane Database Syst. Rev..

[72] Vettoretti S., Molinari P., Armelloni S., Castellano G., Caldiroli L. (2024). Spontaneous low-protein intake in older CKD patients: One diet may not fit all. Front. Nutr..

[73] Yanishi M., Tsukaguchi H., Kimura Y., Koito Y., Yoshida K., Seo M., Jino E., Sugi M., Kinoshita H., Matsuda T. (2017). Evaluation of physical activity in sarcopenic conditions of kidney transplantation recipients. Int. Urol. Nephrol..

[74] Mallamaci F., D’Arrigo G., Tripepi G., Lamberti N., Torino C., Manfredini F., Zoccali C. (2022). Long-Term Effect of Physical Exercise on the Risk for Hospitalization and Death in Dialysis Patients: A Post-Trial Long-Term Observational Study. Clin. J. Am. Soc. Nephrol..

[75] Greenwood S.A., Koufaki P., Mercer T.H., Rush R., O’Connor E., Tuffnell R., Lindup H., Haggis L., Dew T., Abdulnassir L. (2015). Aerobic or Resistance Training and Pulse Wave Velocity in Kidney Transplant Recipients: A 12-Week Pilot Randomized Controlled Trial (the Exercise in Renal Transplant [ExeRT] Trial). Am. J. Kidney Dis..

[76] Kang A.W., Bostom A.G., Kim H., Eaton C.B., Gohh R., Kusek J.W., Pfeffer M.A., Risica P.M., Garber C.E. (2020). Physical activity and risk of cardiovascular events and all-cause mortality among kidney transplant recipients. Nephrol. Dial. Transplant..

[77] van den Ham E.C., Kooman J.P., Schols A.M., Nieman F.H., Does J.D., Akkermans M.A., Janssen P.P., Gosker H.R., Ward K.A., MacDonald J.H. (2007). The functional, metabolic, and anabolic responses to exercise training in renal transplant and hemodialysis patients. Transplantation.

[78] Kanbay M., Copur S., Yildiz A.B., Tanriover C., Mallamaci F., Zoccali C. (2024). Physical exercise in kidney disease: A commonly undervalued treatment modality. Eur. J. Clin. Investig..

[79] Battaglia Y., Baciga F., Bulighin F., Amicone M., Mosconi G., Storari A., Brugnano R., Pozzato M., Motta D., D’alessandro C. (2024). Physical activity and exercise in chronic kidney disease: Consensus statements from the Physical Exercise Working Group of the Italian Society of Nephrology. J. Nephrol..

[80] Zeng D., Ling X.Y., Fang Z.L., Lu Y.F. (2023). Optimal exercise to improve physical ability and performance in older adults with sarcopenia: A systematic review and network meta-analysis. Geriatr. Nurs..

[81] Fiaccadori E., Sabatino A., Schito F., Angella F., Malagoli M., Tucci M., Cupisti A., Capitanini A., Regolisti G. (2014). Barriers to physical activity in chronic hemodialysis patients: A single-center pilot study in an Italian dialysis facility. Kidney Blood Press. Res..

[82] Granak K., Vnucak M., Beliancinova M., Kleinova P., Blichova T., Pytliaková M., Dedinská I. (2024). Regular Physical Activity in the Prevention of Post-Transplant Diabetes Mellitus in Patients after Kidney Transplantation. Medicina.

[83] Sabatino A., Regolisti G., Delsante M., Di Motta T., Cantarelli C., Pioli S., Grassi G., Batini V., Gregorini M., Fiaccadori E. (2019). Noninvasive evaluation of muscle mass by ultrasonography of quadriceps femoris muscle in End-Stage Renal Disease patients on hemodialysis. Clin. Nutr..

[84] Ozturk Z.A., Turkbeyler Ì.H., Abiyev A., Kul S., Edizer B., Yakaryılmaz F.D., Soylu G. (2018). Health-related quality of life and fall risk associated with age-related body composition changes; sarcopenia, obesity and sarcopenic obesity. Intern. Med. J..

[85] Woodle E.S., Gill J.S., Clark S., Stewart D., Alloway R., First R. (2021). Early Corticosteroid Cessation vs. Long-term Corticosteroid Therapy in Kidney Transplant Recipients: Long-term Outcomes of a Randomized Clinical Trial. JAMA Surg..

[86] Zhang P., Fan X., Xiang L., Zhu X., Liu D., Liu J. (2024). Association between physical activity and immunosuppressive medication adherence among renal transplant recipients: A case-control study. BMJ Open.

[87] Johnson J.C., Malik M., Engebretsen T.L., Mujtaba M., Lea A.S., Stevenson H.L., Kueht M.L. (2024). Assessing Long-Term Adverse Outcomes in Older Kidney Transplant Recipients: A Propensity Score-Matched Comparison of Early Steroid Withdrawal Versus Continuous Steroid Immunosuppression Using a Large Real-World Database. Drugs Aging.

[88] Bae S., Chen Y., Sandal S., Lentine K.L., Schnitzler M., Segev D.L., DeMarco M.A.M. (2025). Association of early steroid withdrawal with kidney transplant outcomes in first-transplant and retransplant recipients. Nephrol. Dial. Transplant..

[89] Haller M.C., Royuela A., Nagler E.V., Pascual J., Webster A.C. (2016). Steroid avoidance or withdrawal for kidney transplant recipients. Cochrane Database Syst. Rev..

[90] Zhang L., Rajan V., Lin E., Hu Z., Han H.Q., Zhou X., Song Y., Min H., Wang X., Du J. (2011). Pharmacological inhibition of myostatin suppresses systemic inflammation and muscle atrophy in mice with chronic kidney disease. FASEB J..

[91] Verzola D., Barisione C., Picciotto D., Garibotto G., Koppe L. (2019). Emerging role of myostatin and its inhibition in the setting of chronic kidney disease. Kidney Int..

[92] Rooks D., Swan T., Goswami B., Filosa L.A., Bunte O., Panchaud N., Coleman L.A., Miller R.R., Garayoa E.G., Praestgaard J. (2020). Bimagrumab vs. Optimized Standard of Care for Treatment of Sarcopenia in Community-Dwelling Older Adults: A Randomized Clinical Trial. JAMA Netw. Open.

[93] Suh J., Lee Y.S. (2020). Myostatin Inhibitors: Panacea or Predicament for Musculoskeletal Disorders?. J. Bone Metab..

[94] Rooks D., Praestgaard J., Hariry S., Laurent D., Petricoul O., Perry R.G., Lach-Trifilieff E., Roubenoff R. (2017). Treatment of Sarcopenia with Bimagrumab: Results from a Phase II, Randomized, Controlled, Proof-of-Concept Study. J. Am. Geriatr. Soc..

[95] Becker C., Lord S.R., Studenski S.A., Warden S.J., Fielding R.A., Recknor C.P., Hochberg M.C., Ferrari S.L., Blain H., Binder E.F. (2015). Myostatin antibody (LY2495655) in older weak fallers: A proof-of-concept, randomised, phase 2 trial. Lancet Diabetes Endocrinol..

[96] Wagner K.R., Fleckenstein J.L., Amato A.A., Barohn R.J., Bushby K., Escolar D.M., Flanigan K.M., Pestronk A., Tawil R., Wolfe G.I. (2008). A phase I/IItrial of MYO-029 in adult subjects with muscular dystrophy. Ann. Neurol..

[97] Carrero J.J., Stenvinkel P., Cuppari L., Ikizler T.A., Kalantar-Zadeh K., Kaysen G., Mitch W.E., Price S.R., Wanner C., Wang A.Y. (2013). Etiology of the protein-energy wasting syndrome in chronic kidney disease: A consensus statement from the International Society of Renal Nutrition and Metabolism (ISRNM). J. Ren. Nutr..

[98] Fahal I.H. (2014). Uraemic sarcopenia: Aetiology and implications. Nephrol. Dial. Transplant..

[99] Delgado C., Doyle J.W., Johansen K.L. (2013). Association of frailty with body composition among patients on hemodialysis. J. Ren. Nutr..

